# Diagnostic behaviour of general practitioners when suspecting Lyme disease: a database study from 2010-2015

**DOI:** 10.1186/s12875-018-0729-2

**Published:** 2018-04-03

**Authors:** Esmée Botman, C. Wim Ang, Johanna H. K. Joosten, Pauline Slottje, Johannes C. van der Wouden, Otto R. Maarsingh

**Affiliations:** 1 Department of General Practice & Elderly Care Medicine and Amsterdam Public Health research institute, VU University Medical Center, Van der Boechorststraat 7, 1081 BT Amsterdam, The Netherlands; 2 Department of Medical Microbiology & Infection Control, VU University Medical Center, Amsterdam, The Netherlands; 3 Academic Network of General Practice, Department of General Practice & Elderly Care Medicine, VU University Medical Center (ANH VUmc), Amsterdam, The Netherlands

**Keywords:** Lyme disease, Diagnosis, General practice, Primary health care

## Abstract

**Background:**

Due to the raised public awareness of Lyme Borreliosis (LB), its increased incidence and the increased availability of serological tests, the demand for diagnostic testing on LB has increased. This may affect the diagnostic behaviour of general practitioners (GPs). Aim of our study was to describe GPs’ diagnostic behaviour when suspecting LB.

**Methods:**

In this descriptive study from January 2010 to June 2015, we used the anonymized electronic medical records of 56,996 patients registered in 12 general practices in Amsterdam, The Netherlands. The target population was identified by means of an extensive search strategy, based on International Classification of Primary Care (ICPC-1) codes, free text and diagnostic test codes. All contacts related to LB were included in the analysis.

**Results:**

2311 patients were included, accounting for 3861 LB contacts and 2619 LB episodes. The distribution of LB contacts showed annual peaks during spring and summer. Serological testing was performed in 36.4% of LB episodes and was mostly requested in patients presenting with general symptoms (71.4%). Unnecessary testing often occurred and only 5.9% of the tests turned out to be positive by immunoblot. From January 2010 to June 2015, no significant differences were found in the number of requested serological tests. The level of serological testing during LB episodes differed significantly between the general practices (19.2% to 75.8%).

**Conclusions:**

Contrary to clinical guidelines, GPs regularly requested serology even when there was a low suspicion of LB. The development of an easy-to-use diagnostic algorithm may decrease overuse of diagnostic tests and thereby reduce overtreatment of LB.

## Background

Lyme Borreliosis (LB) is the most common tick-borne disease in the world [1–4] caused by *Borrelia burgdorferi* sensu lato-spirochetes, of which *Borrelia afzellii* and *Borrelia garinii* are most prevalent in Europe [1, 5]. Over the past decades, the incidence of LB in Europe has increased to approximately 65,500 patients per year [6–9]. Raised public awareness of LB, its various clinical manifestations, its treatable character, the fear of disease among patients and the increased availability of serological tests have driven a rising demand for diagnostic testing [8, 10, 11]. Current European guidelines, although not tailored to primary care, clearly state that serological tests must only be done when there is a high suspicion of LB. This high a priori probability is mainly based on LB-specific symptoms (like symptoms suggesting Lyme arthritis or neuroborreliosis) [12, 13]. Requesting non-indicated tests may lead to false-positive results, since - depending on the assay - 3-9% of healthy controls is seropositive for Borrelia antibodies [14]. This is because serological tests do not differentiate between an active LB and an (asymptomatic) infection from the past [11]. Other reasons for a false-positive test result are infections with other related pathogens (e.g. syphilis), autoimmune disorders, or cross-reactivity between spirochetes. [15] In general, according to Bayes’ theorem, the usefulness of a serological test for LB depends on the pre-test probability and the subsequent predictive values in the setting where the test is being used [2, 16, 17]. However, even in a tertiary, multidisciplinary setting it is challenging to either rule out or demonstrate an association with Borrelia burgdorferi sensu lato. [18] The seropositivity rates for LB vary throughout Europe, from 3.4% in the general population in Italy to 15.2% in France [14, 19, 20]. When testing high-risk populations, these seropositive rates will be even higher. In Poland, healthy forestry workers reach a seropositivity rate of 45% [19, 21]. Because positive test results are often interpreted as Borrelia being the causal agent of the symptoms, overtreatment is common in cases of false-positive test results. Previous studies in general practices have only focused on tick bites and erythema migrans (EM). In Belgium 50% of the patients presenting with a typical EM got serologically tested by the GP [9], contrary to national/international guidelines (i.e. patients presenting with EM should always receive antibiotic treatment, so serological testing has no additional value; moreover, a false negative test result may occur, which can be misleading [12, 13].

The aim of the present study was to describe GPs’ diagnostic behaviour with respect to LB. Our study is the first to describe seasonal trends in LB contacts, to link symptoms to serology requests, and to investigate if GPs’ diagnostic behaviour on LB has changed over the years 2010-2015.

## Methods

**Fig. 1:**
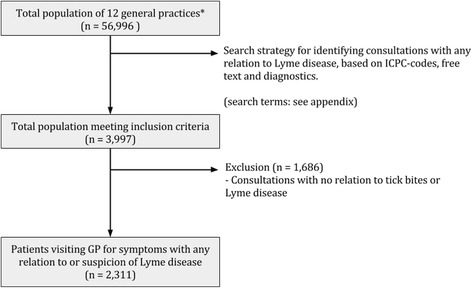
Flowchart of the identification of general practice contacts related to Lyme Borreliosis. * Based on registered patients in 2014. GP: General Practitioner; ICPC-1 = International Classification of Primary Care

An observational study was carried out with anonymized data extracted from the database of the Academic Network of General Practice of VU University Medical Center (ANH VUmc), Amsterdam. The database contains pseudonimized primary health care data. We used the anonymized data of 56,996 patients registered in 12 general practices (with more than 60 GPs) in Amsterdam, from January 2010 to June 2015. In the Netherlands, almost all non-institutionalised citizens are registered to a general practice and the GP acts as gatekeeper. We developed an extensive search and selection strategy, based on the International Classification of Primary Care (ICPC-1) codes, free text and diagnostic test codes, to identify contacts related to LB (see Fig. 1 for the flow chart, Appendix 1 for the search strategy, and Appendix 2 for the origin of the identified patients, divided by search strategy). Annotations of the GPs from all identified contacts (consultations, telephone, home visits, e-mails) were reviewed by one of the investigators (EB). Contacts not related to LB or tick bites were excluded (e.g. other insect bites). A random selection of 5% of the identified contacts was reviewed by a second, independent reviewer to check the reliability of the data extraction. Disagreements were resolved during a consensus meeting between investigator and independent reviewer.

The following data were extracted from the selected medical records (analytical sample): basic characteristics of the patients (i.e. sex, age at the time of the first LB contact), characteristics of the LB contact and episode (i.e. date of contact, type of contact, number of contacts), ICPC-1 code, symptoms registered, findings during physical examination, diagnosis and management (i.e. Lyme serology or referral). Contacts belonging to the same episode were merged for the analysis of presenting symptoms and diagnostic testing. In the analysis, a distinction was made between individual contacts and episodes that include all contacts concerning the same complaint. Also, a distinction was made between a definite tick bite and a possible tick bite; the latter was called ‘insect sting’ in the analysis.

The data contained different Lyme serology tests from different laboratories and it was not possible to identify the exact assay that was used for each patient. Based on the quantitative value and the cut-off level we could infer that the majority of the samples were screened with the Immunetics C6 ELISA kit (Immunetics Inc., Boston, USA). We used the available laboratory interpretation of the screening test. This included the requirement that all equivocal or positive screening test results had to be confirmed by an immunoblot. Laboratories classified immunoblot outcomes as ‘negative’, ‘inconclusive’ or ‘positive’.

Descriptive analysis was performed using SPSS 23.0. Chi-square tests for trend (linear-by-linear) were used for comparing differences between years regarding contacts, episodes and serological tests.

The ANH database is run according to Dutch privacy legislation and contains pseudonymized general practice care data from all patients of the participating general practices, excluding those patients who object to this. Observational studies based on anonymized data from the ANH VUmc database are exempted from informed consent of patients.

## Results

Figure 1 provides a flowchart of the study. Twelve general practices were included (*N* = 56,996 patients), resulting in 2311 patients who visited the GP because of complaints or concerns related to LB in the period January 2010 to June 2015 (see Appendix 3 for practice characteristics). The reliability of the data extraction was acceptable: from a random selection of 5% of identified LB contacts, 6 out of 350 contacts (1.7%) had been classified incorrectly.

The basic characteristics of patients and their LB contacts are shown in Table 1.

**Table 1:** Characteristics of patients and general practice contacts related to Lyme Borreliosis

Variables	Number	Percent
Patients (*N* = 2311)		
Gender, female	1371	59.3
Age, mean (95% CI)^a^	39.65 (38.85-40.52)	
≤ 18	389	16.8
19-64	1639	70.9
≥ 65	283	12.2
Contacts (*N* = 3681)		
Type of contact		
Consultations	2796	76.0
Telephone	869	23.6
Home visits	12	0.3
E-mail	4	0.1
Year of contact		
2010	635	17.3
2011	715	19.4
2012	548	14.9
2013	694	18.9
2014	740	20.1
2015^b^	349	9.5

*CI* Confidence Interval

^a^Age at time of the first contact of an episode

^b^Only first six months available

**Fig. 2:**
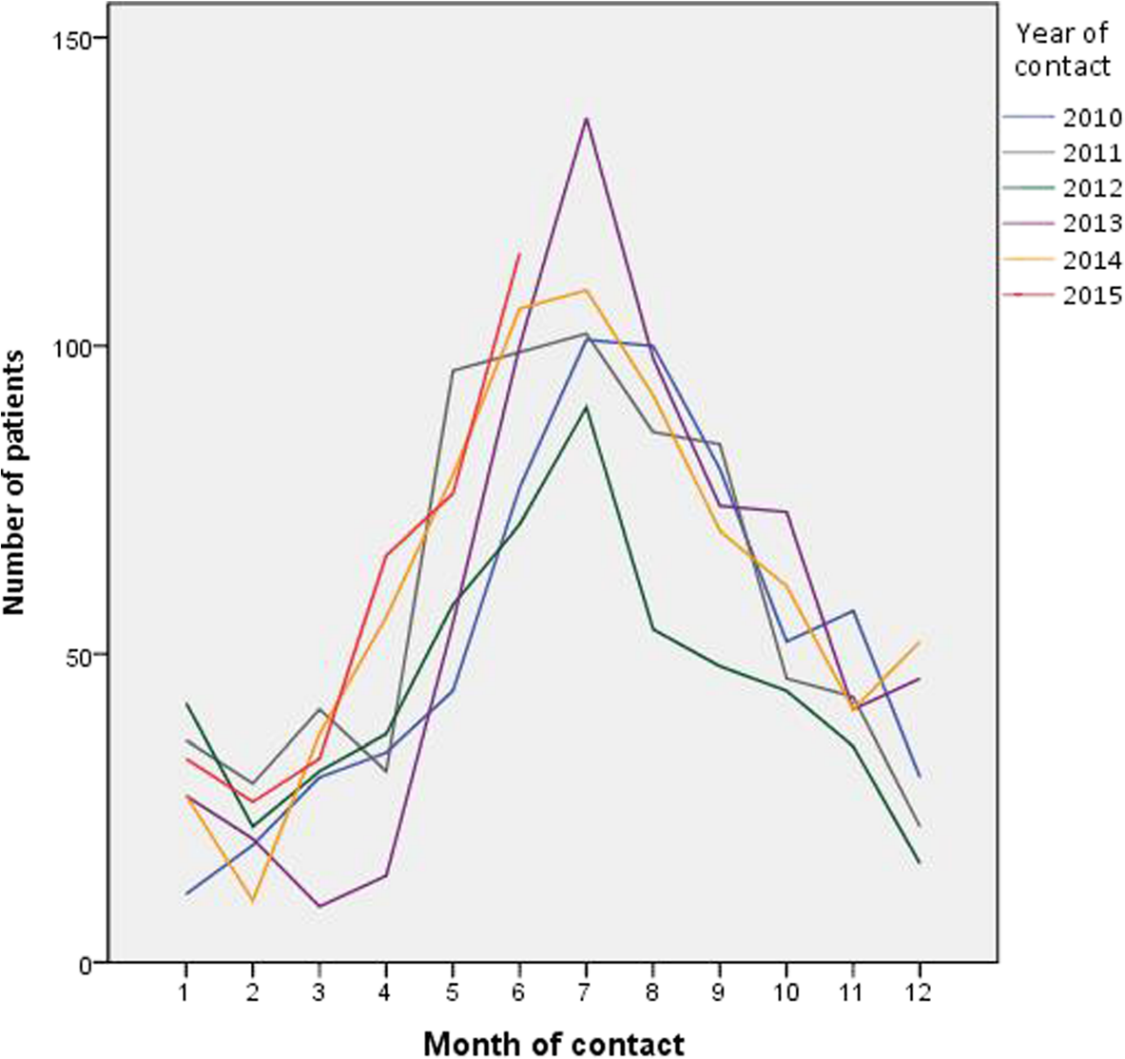
Distribution of general practice contacts related to Lyme Borreliosis throughout the year* * Contacts include consultations, visits, calls and e-mails. Data of 2015 were available until June

The overall incidence of episodes related to LB was 8.8 per 1000 person years within the dataset of 56,996 patients. The incidence of LB episodes across the years 2010-2014 did not show a significant change (*p* = 0.932). Since data for 2015 were only partially available, 2015 was not included in this analysis. Each year, LB related contacts showed a peak during spring and summer (May until September; Fig. 2). The majority of patients (78.2%) contacted their GP only once within an LB episode. For LB episodes with 2 or more contacts, the mean interval between first and last contact was 45 days. Most patients (88%) experienced only one LB episode over the period January 2010 to June 2015.

Table 2 provides an overview of reasons for encounter during episodes related to LB (*N* = 2619 episodes). Also, the table illustrates per reason for encounter how often serological testing was ordered and how often this resulted in a positive immunoblot test result.

**Table 2:** Reasons for encounter registered in electronic medical record for episodes related to Lyme Borreliosis (*N* = 2619 episodes)

Registered reasons for encounter (during *N* = 2619 episodes)				Serological Testing		Positive immunoblot test result	
	*N*	%^c^		*N*	%^c^	*N*	%^c^
I. General symptoms							
Fatigue	458	17.5		357		20	
Malaise	162	6.2		89		4	
Gastro-intestinal	74	2.8		59		5	
Respiratory	39	1.5		34		3	
Other	233	8.9		151		12	
Total	966	36.9		690	71.4	44	6.4
II. Neurological symptoms							
Headache	105	4.0		72		6	
Dizziness	64	2.4		51		4	
Pain with radiation	50	1.9		34		6	
Numbness	41	1.6		26		4	
Paresthesia	47	1.8		26		2	
Other	96	3.7		70		5	
Total	403	15.4		207	51.4	27	13.0
III. Musculoskeletal symptoms							
Arthralgia	240	9.2		120		15	
Myalgia	92	3.5		61		4	
Total	332	12.7		181	54.5	19	10.5
IV. Diagnostic and therapeutic request^d^							
Serology request	196	7.5		154		18	
Referral request	31	1.2		15		5	
Antibiotics request	60	2.3		10		1	
Total	287	11.0		179	62.4	24	13.4
V. Psychological							
Fear of Lyme	434	16.6		146		14	
Other	27	1.0		23		1	
Total	461	17.6		169	36.7	15	8.9
VI. Skin abnormality							
Possible erythema migrans	120	4.6		34		6	
Local irritation tick bite	327	12.5		33		2	
Erythema migrans	136	5.2		25		7	
Sting bite	194	7.4		12		0	
Eczema	35	1.3		8		2	
Dermatomycosis	65	2.5		7		0	
Other	203	7.8		33		5	
Total	1080	41.2		152	14.1	22	14.5
VII. Recent insect sting^a^							
Tick bite	914	34.9		121		5	
Insect bite	248	9.5		36		3	
Total	1162	44.4		157	13.5	8	5.1
Total^b^	4691	179.1					

^a^Recent is defined as a sting within 3 months prior to contact

^b^Adds up to more than 100%, because general practitioners may register more than one reason for encounter per episode

^c^Row percentages

^d^Request directly made by the patient

Symptoms most frequently registered by GPs in LB episodes were recent insect sting, defined as ‘recent’ when stung within three months prior to contact (*N* = 1162, 44.4% of episodes), skin abnormalities (*N* = 1080, 41.2%) and general symptoms like fatigue and malaise (*N* = 966, 36.9%; Table 2).

Lyme serology was requested during 953 out of 2619 episodes (36.4%). GPs mostly requested serology for episodes in which patients presented with general symptoms (*N* = 690, 71.4%). The percentage of positive test results was highest for the episodes in which patients presented with skin abnormalities (14.5%) and for episodes in which patients requested for a test themselves (13.4%; Table 2). GPs requested serology for 18.4% (*N* = 25 out of 136) of the episodes in which patients presented with typical EM, for 10.1% (*N* = 33 out of 327) of the episodes in which patients presented with a locally irritated tick bite, and for 13.2% (*N* = 121 out of 914) of the episodes in which patients presented with an asymptomatic tick bite.

From January 2010 to June 2015, the number of requested serological tests did not change significantly (*p* = 0.190). Of the 953 serological tests, 19.3% was screened positively. Only 5.9% of these were confirmed by the immunoblot. Referral to a specialist occurred in 7.6% of all LB episodes, mostly to neurologists (24.2%) or internists (21.7%; Table 3).

**Table 3:** Referral to specialists for episodes related to Lyme Borreliosis (*N* = 2619 episodes)

Referral	Number	Percent
Non-referred	2421	92.4
Referred	198	7.6
Neurologist	48	24.2
Internist	43	21.7
Dermatologist	22	11.0
Lyme Centre^a^	16	8.1
Rheumatologist	12	6.1
Paediatrician	8	4.0
Other^b^	49	24.2
Total	2619	100.0

^a^Multidisciplinary centre specialized in Lyme Borreliosis

^b^E.g. cardiologist, rehabilitation specialist, and psychologist

**Fig. 3:**
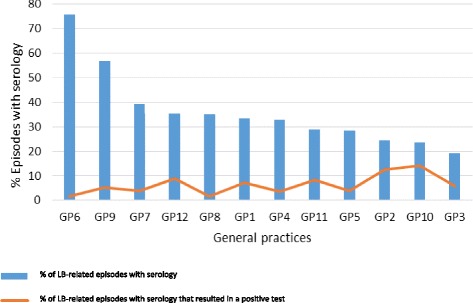
Serology requests and positive test rate in LB-related episodes per general practice

The general practices showed considerable differences with respect to serological testing during LB episodes, ranging from 19.2% to 75.8% of the LB episodes. There was a tendency for practices with lower serological testing rates to have higher positive tests rates (Fig. 3).

## Discussion

### Summary of main findings

This study was performed to describe GPs’ diagnostic behaviour with respect to LB. As expected, LB contacts showed annual peaks during spring and summer. Of all episodes, 36.4% were followed by a serological test for LB. Tests were mainly performed for episodes in which patients presented with nonspecific complaints like fatigue and headache. Contrary to clinical guidelines, a serological test was performed in 18.4% of the episodes with a typical EM, 10.1% of the episodes with a locally irritated tick bite, and 13.2% of the episodes with an asymptomatic tick bite. Overall, only 5.9% of the serological tests turned out to be positive which is as high as the seropositivity rate of LB in the general population [14]. Also, considerable differences between the 12 general practices were found regarding the rates of serological requests. The number of LB contacts and requested serological tests did not change over the period January 2010 to June 2015.

### Strengths and weaknesses

This study is the first to explore seasonal trends in LB contacts, to link symptoms with serology requests, and to investigate time trends in GPs diagnostic behaviour on LB over a period of four-and-a-half years. A strength of our study is the use of an extensive, combined search strategy (see Appendix 1). Such a search strategy is crucial, as single search strategies – based on either free text, ICPC-1 code, or diagnostic testing code – all would miss a significant proportion of the target population (see Appendix 2). For example, if one would not use the free text search, 610 patients (26.4% of all 2311 identified patients) and 1023 contacts (27.8% of all 3681 identified contacts) would have been missed. This would have led to an overestimation of the level of serological testing during LB related episodes. Some potential limitations need to be considered. Firstly, the quality of the data depends on the completeness and accuracy of registration by the GP and our selection and data extraction strategy. Relevant contacts may have been missed, which would then have resulted in an underestimation of the actual contact frequency for LB. However, we expect this to be unlikely, because we did not only rely on ICPC-1 codes but also included the free text annotations - which were mined on relevant search terms - and diagnostic testing codes. In addition, GPs affiliated to the ANH VUmc database receive regular training in EMR coding and registration. Secondly, episodes in which both patient and GP did not suspect nor mention LB were not included. Therefore, we were not able to measure underdiagnosis, i.e. not requesting a test although indicated. Finally, we studied data from general practices in an urban area, which may limit the generalizability to rural areas, or other (European) areas, given the reported increasing gradient from west to east (highest in central-eastern Europe), and decreasing gradient from south to north in Scandinavia and from north to south in Italy, Spain and Greece [20].

### Comparison with existing literature

The seropositive prevalence of LB in the general Dutch population is 5-10% [11, 12, 14, 22]. Given the low percentage of seropositive patients (5.9%) in our study population, it is likely that GPs regularly requested serology when there only was a low suspicion on LB [23]. This is substantiated by the finding that GPs mostly requested serology for episodes in which patients presented with general/nonspecific symptoms. According to the current guidelines, EM and asymptomatic tick bites should not be followed by a serological test on LB [12, 13]. Nevertheless, the GPs in our sample performed serological testing in one fifth of all patients with EM. This may seem substantial, but other international studies present much higher percentages, i.e. 50% and 68% in Belgium and France, respectively [9, 24]. Also, 121 out of 914 (13.2%) asymptomatic tick bites were serologically tested. In Belgium, 17.5% of the asymptomatic tick bites got serologically tested [9].

In our study, serology was mostly requested for episodes in which patients presented with nonspecific symptoms. Previous studies showed that unexplained symptoms, other than LB, more frequently lead to laboratory testing by the GP [25, 26]. Reasons for ordering tests for patients with unexplained symptoms may be a limited tolerance to diagnostic uncertainty or time pressure [25]. Once a laboratory test has been requested, the threshold for more testing is low and many GPs are unaware of the consequences (like false positive findings) [25, 26]. For example, an Australian study showed that 64.2% of patients presenting with fatigue to their GP received laboratory testing. In only 4% of the patients the tests led to a significant clinical diagnosis [26]. Improving GPs’ knowledge on the subject, combined with an easy-to-use diagnostic algorithm may increase confidence and reduce the overuse of diagnostic tests.

The large differences in serological testing between general practices suggest that - despite multidisciplinary guidelines - there is no consensus how to apply these guidelines in daily general practice and when to perform serological testing on LB.

### Implications for clinicians and research

Hopefully, our study will initiate the development of an easy-to-use diagnostic algorithm for LB that is (also) applicable in daily general practice. Although the current scientific evidence for such an algorithm is limited, some promising potential predictors have been reported, like tick engorgement and patient-estimated duration of tick attachment. [10, 27] Future research may focus on the needs and expectations of patients and GPs during LB consultations, for example by a focus group study or semi-structured interviews. This may reveal clues to arrive at optimal (diagnostic) healthcare decisions with respect to LB.

## Conclusions

This descriptive GP database study, covering the period January 2010 to June 2015, was the first to investigate seasonal trends in LB contacts, to link symptoms to serology requests, and to investigate GPs’ diagnostic behaviour over time. The distribution of LB contacts showed annual peaks during spring and summer. Episodes in which patients presented with general symptoms, like fatigue, were most frequently followed by serological testing. Serological testing was performed in 36.4% of LB episodes; only 5.9% of these tests turned out to be positive. The high number of LB consultations with nonspecific complaints together with the low frequency of positive serological tests, and the considerable inter-practice differences indicate a high number of inappropriate tests and underscores the need for an easy-to-use diagnostic algorithm that is (also) applicable in daily general practice.

## Appendix 1

**Table 4:** Search strategy in electronic medical records

Search term or code	Explanation
I. Free text	
Teek	Tick
Teke^b^	Tick
Lyme	Lyme Borreliosis
Ery^b^Migr^b^	Erythema migrans
EM	Erythema migrans
E.M.	Erythema migrans
Bor^b^elia	Lyme Borreliosis
II. ICPC-1 code	
S12	Insect sting
S12.01	Tick bite
A78	Other infectious diseases
A78.05	Lyme Borreliosis
III. Diagnostic test code^a^	
125	Borrelia Burgdorferi antibodies IgA
126	Borrelia B. confirmation test
127	Borrelia Burgdorferi antibodies IgG
2889	Borrelia Burgdorferi antibodies IgG liquor
128	Borrelia Burgdorferi antibodies
2404	Borrelia Burgdorferi index per total antibodies IgG/IgM
129	Borrelia Burgdorferi IgM
3104	Borrelia Burgdorferi antibodies IgM (1e mnt,gprd)
3434	Borrelia Burgdorferi antibodies IgM (quantitative)
2891	Borrelia Burgdorferi antibodies IgM liquor
2167	Borrelia Burgdorferi C6 protein
3101	Borrelia Burgdorferi C6 protein (quantitative)
3102	Borrelia Burgdorferi C6 protein (1e mnt, gprd)
2890	Borrelia Burgdorferi C6 protein liquor
2226	Borrelia Burgdorferi antibodies IgG (Westernblot)
2227	Borrelia Burgdorferi antibodies IgM (Westernblot)

Our search strategy was based on selected International Classification Primary Care (ICPC-1) Codes, words in free text annotations, and requests for serological tests for Lyme disease as recorded in routine primary care in the electronical medical records

*ICPC-1* International Classification of Primary Care

^a^Tests were included when requested 8 days before or after a contact

^b^Wild card code, to include any variation in typing following trunk

## Appendix 2

**Table 5:** Origin of identified patients and general practice contacts, divided by search strategy

Identified patients (*N* = 2311)				
Search strategy			Number of patients	%
I. Free text	II. ICPC-1 code	III. Diagnostic test code		
X	–	–	610	26.4
–	X	–	62	2.7
–	–	X	331	14.3
X	X	–	801	34.7
–	X	X	4	0.2
X	–	X	325	14.1
X	X	X	178	7.7
Total			2311	100
Identified general practice contacts (*N* = 3681)				
Search strategy			Number of contacts	%
I. Free text	II. ICPC-1 code	III. Diagnostic test code		
X	–	–	1023	27.8
–	X	–	182	4.9
–	–	X	797	21.7
X	X	–	1093	29.7
–	X	X	40	1.1
X	–	X	427	11.6
X	X	X	119	3.2
Total			3681	100

ICPC-1 = International classification of Primary care

X: Used as search term to identify patient and contacts, respectively

-: Not used in search

## Appendix 3

**Table 6:** Characteristics of the general practices (*N* = 12)

General practice	Type of practice^a^	Number of patients per practice (annual mean)		Number of GPs
		*2010*	*2015*	
1.	Group	4531	5562	4
2.	Solo	2684	2717	1
3.	Group	4432	4705	8
4.	Duo	2848	3351	2
5.	Duo	2663	2887	2
6.	Duo	1881	2094	2
7.	Group	3284	5808	5
8.	Duo	2777	2949	2
9.	Group	8812	8984	5
10.	Group	4852	4763	4
11.	Group	8075	7870	6
12.	Group	5499	6390	4

^a^: Solo (1 GP), duo (2 GPs), or group (> 2 GPs)

## Abbreviations

ANH VUmc: Academic Network of General Practice of VU University Medical Center
CI: Confidence Interval
EM: Erythema migrans
GP: General Practitioner
ICPC-1: International Classification of Primary Care
LB: Lyme Borreliosis
